# Processing of X-ray snapshots from crystals in random orientations

**DOI:** 10.1107/S1399004714013534

**Published:** 2014-07-25

**Authors:** Wolfgang Kabsch

**Affiliations:** a Max-Planck-Institut für medizinische Forschung, Jahnstrasse 29, D-69120 Heidelberg, Germany

**Keywords:** *nXDS*

## Abstract

A functional expression is introduced that relates scattered X-ray intensities from a still or a rotation snapshot to the corresponding structure-factor amplitudes. The new approach was implemented in the program *nXDS* for processing monochromatic diffraction images recorded by a multi-segment detector where each exposure could come from a different crystal. For images containing indexable spots, the intensities of the expected reflections and their variances are obtained by profile fitting after mapping the contributing pixel contents to the Ewald sphere. The varying intensity decline owing to the angular distance of the reflection from the surface of the Ewald sphere is estimated using a Gaussian rocking curve. This decline is dubbed ‘Ewald offset correction’, which is well defined even for still images. Together with an image-scaling factor and other corrections, an explicit expression is defined that predicts each recorded intensity from its corresponding structure-factor amplitude. All diffraction parameters, scaling and correction factors are improved by post-refinement. The ambiguous case of a lower point group than the lattice symmetry is resolved by a method reminiscent of the technique of ‘selective breeding’. It selects the indexing alternative for each image that yields, on average, the highest correlation with intensities from all other images. Processing a test set of rotation images by *XDS* and treating the same images by *nXDS* as snapshots of crystals in random orientations yields data of comparable quality, clearly indicating an anomalous signal from Se atoms.

## Abstract 2

A new method for the treatment of partial reflections from X-ray snapshots is implemented in the program package *nXDS*, which yields intensity data of almost the same quality as those obtained by the classical rotation method.

## 1. Introduction   

The availability of free-electron lasers (FELs) as a source of ultrabright X-ray pulses of femtosecond duration has provided a new approach for collecting diffraction data that makes extremely small and radiation-sensitive samples accessible to structural studies. It is expected that this new X-ray source will strongly contribute to our knowledge of the large group of biological objects such as membrane proteins that are difficult to grow as macroscopic crystals.

Destruction of the irradiated object by a single pulse is much slower than the pulse duration, so that data can be collected at room temperature before the sample deteriorates owing to radiation damage. As a consequence many diffraction snapshots are required, each from a different sample in a random orientation, to yield a complete data set. For crystalline samples diffraction data are partially recorded on still images, which presents a challenging task to processing that traditional crystallographic programs were not designed to handle.

This has led to the development of new software suites: (i) *CrystFEL* (White *et al.*, 2012 ▶, 2013 ▶), which uses *MOSFLM* (Leslie & Powell, 2007 ▶; Powell *et al.*, 2013 ▶) or *DirAx* (Duisenberg, 1992 ▶) for reflection indexing and merges individual intensity measurements (Kirian *et al.*, 2010 ▶, 2011 ▶) to estimate the integrated intensity of each unique reflection, and (ii) the *cctbx.xfel* (Sauter *et al.*, 2013 ▶; Hattne *et al.*, 2014 ▶) and *cctbx.spotfinder* (Zhang *et al.*, 2006 ▶) components within the *Computational Crystallography Toolbox* (*cctbx*). These programs rely on the Monte Carlo method for estimating reflection intensities, thereby assuming that other factors such as fluctuations in the incident-beam intensity, wavelength and spectrum as well as the irradiated volume of the specimen ‘integrate out’ upon averaging (White *et al.*, 2012 ▶; Kirian *et al.*, 2011 ▶). It has recently been demonstrated (Barends *et al.*, 2014 ▶) that a *de novo* crystal structure can be determined from X-ray free-electron laser data analyzed by *CrystFEL*. The data comprise about 60 000 indexed images out of 2.4 million snapshots from a lysozyme heavy-atom derivative that gives a strong anomalous signal from two Gd atoms per asymmetric unit (Barends *et al.*, 2014 ▶).

Another complication that is not encountered in traditional data collection by the rotation method is the ambiguity in the choice of unit-cell basis vectors in cases where the lattice symmetry is higher than the crystal symmetry. The problem arises because the reflections recorded by each snapshot are indexed independently and their partial intensities are too inaccurate for any decision-making based on correlations involving different exposures (White *et al.*, 2012 ▶). To circumvent this problem, *CrystFEL* generates a perfectly twinned data set of higher symmetry by merging all snapshots, which renders the subsequent structure solution more difficult. Recently, new methods for breaking the indexing ambiguity have been described (Brehm & Diederichs, 2014 ▶) and the detour *via* artificially twinned data sets appears to no longer be necessary.

Some of the problems in processing FEL snapshots had already been encountered more than 35 years ago when trying to solve the first virus crystal structures. Often, on account of radiation damage, the crystal had to be replaced after a single exposure covering only a small rotation range. This resulted in many partially recorded reflections and their complete intensities had to be estimated. A solution to this problem, the ‘post-refinement’ method, was developed (Schutt & Winkler, 1977 ▶; Rossmann *et al.*, 1979 ▶; Harrison *et al.*, 1985 ▶; Rossmann, 1985 ▶) to derive complete intensities from refined estimates for the fractions of observed intensity, the ‘partiality’. For rotation images the partiality of each reflection can always be calculated as a function of orientation, the unit-cell metric, the mosaic spread of the crystal and a model of the reflection profile. The idea of the ‘post-refinement’ method is to improve these parameters after all images have been processed by using symmetry-related, fully recorded reflections for reference.

As an alternative to the processing of snapshots by Monte Carlo integration, the software package *nXDS* has been developed that resumes the old ‘post-refinement’ idea, now enriched by a model for the recorded snapshot intensities as a function of their corresponding structure-factor amplitudes and also including the correct treatment of stills. The unique reflection intensities and diffraction parameters for all crystals are refined simultaneously to minimize the discrepancy between observed and expected spot centroids and recorded intensities. The indexing ambiguity is resolved by a method reminiscent of ‘selective breeding’. It selects the indexing alternative for each image that yields, on average, the highest correlation with symmetry-related reflection intensities from all other images. The implementation of *nXDS* is derived from the standard *XDS* package (Kabsch, 2010*a* ▶,*b* ▶) in its present version, which includes the handling of multi-segment detectors.

## 2. Description of the diffraction experiment   

Any right-handed orthonormal *laboratory* coordinate system {**l**_1_, **l**_2_, **l**_3_} can be chosen with its origin at the intersection point of the beam and the crystal. This reference system serves to specify the following.

(i) The monochromatic incident-beam wavevector **S**_0_ (wavelength λ, |**S**_0_| = 1/λ) pointing from the X-ray source towards the crystal.
(ii) The fixed rotation axis **m**_2_ passing through the origin of the *laboratory* system. During X-ray exposure the crystal is uniformly rotated by some angle Δ_ϕ_ around this axis. Still images are treated as a limiting case where Δ_ϕ_ = 0 and the concept of a rotation axis loses its meaning.
(iii) The right-handed set of unit-cell basis vectors {**b**_1_, **b**_2_, **b**_3_} of the single crystal and the associated reciprocal basis 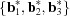 such that any reciprocal-lattice point can be expressed as 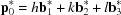 where *h*, *k*, *l* are integers.
(iv) The right-handed orthonormal *detector* system {**D**_1_, **D**_2_, **D**_3_} imagined to be fixed in the instrument and translated by the origin vector **D**_0_.

The detector consists of one or several rectangular, planar X-ray-sensitive segments. Their position and orientation is specified with respect to the *detector* system, which renders the specification independent of any detector movements. For a well calibrated instrument this greatly reduces the number of parameters that need to be refined during data processing.

A right-handed orthonormal *segment* system {**d**′_1_, **d**′_2_, **d**′_3_} and an origin vector **d**′_0_ are specified for each segment with respect to the *detector* coordinate system. The X-ray-sensitive area consists of identical pixels of sizes *Q*_*X*_, *Q*_*Y*_ (mm) along **d**′_1_ and **d**′_2_, respectively.

Expressing the segment origin **d**′_0_ in terms of {**d**′_1_, **d**′_2_, **d**′_3_}, the location **x**′ of a segment pixel *x*, *y* with respect to the *detector* system is given by 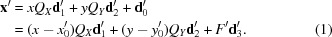
Thus, the *laboratory* coordinates *x* of the same pixel *x*, *y* are 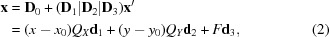
where 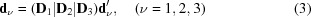
and 
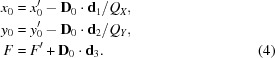
The segment plane is found at a distance |*F*| from the crystal. *F* has a negative sign if **d**_3_ points towards the crystal.

For accurate integration, the spot shape and extent are modelled as Gaussian distributions involving two parameters: the standard deviations of the reflecting range (mosaicity), σ_M_, and the combined effects of beam divergence and mosaicity, σ_D_.

## 3. Processing steps of *nXDS*   

The program package *nXDS* was developed for automatic determination of scaled and fully corrected reflection intensities from monochromatic X-ray diffraction images, each from a different crystal in a random orientation. *nXDS* uses many ideas, routines and the overall structure of the rotation data-processing program *XDS* (Kabsch, 2010*a* ▶,*b* ▶) and includes numerous new routines for the efficient evaluation of a large number of snapshots. The algorithms developed for integration, scaling and post-refinement are described in the following.

The snapshots are processed by *nXDS* in seven steps, named *XYCORR*, *INIT*, *COLSPOT*, *POWDER*, *IDXREF*, *INTEGRATE* and *CORRECT*, which are called in succession by *nXDS*. Information is communicated between the steps by files, which allows the repetition of selected steps with a different set of input parameters without rerunning the whole program.

### 3.1. *XYCORR*   

If necessary for the detector being used, this step computes lookup tables of spatial corrections for each detector pixel. In subsequent data-processing steps, when the true coordinates of a pixel with respect to the laboratory coordinate system are needed, the correction values for the *X* and *Y* coordinates are retrieved from the tables and added to the pixel’s array coordinates in the data image. This step is essentially the same as that used by *XDS*.

### 3.2. *INIT*   

Three lookup tables are determined here that are required by the subsequent processing steps for classifying pixels in the data images as untrusted, background or belonging to a diffraction spot. This step is virtually the same as that used by *XDS*.

### 3.3. *COLSPOT*   

Strong diffraction spots occurring in the data images are located and their centroids are saved in a file. Compared with the corresponding step in *XDS* a simplification resulted for snapshots from the fact that there are no neighbouring images with contributions to the same spot.

### 3.4. *POWDER*   

The origin of the *detector* system can often be found from a powder pattern generated from the spots located in the previous step. An incorrectly specified origin could lead to a misindexing of reflections, a risk that is particularly high for processing snapshots. As the use of multi-segment detectors can prevent direct recognition of the powder circles, the following method was devised.

A plane with the incident-beam vector **S**_0_ as its normal is constructed from a second vector **t** = (1, 1, 1). To assure that **t** is non-collinear with **S**_0_, one of its components is reset to 0, namely the component corresponding to the maximum absolute value of the components of **S**_0_. If this is not unique, the first occurrence of the maximum is taken. For example, **t** = (1, 0, 1) if the *y* coordinate is the first occurrence of the absolutely largest component of **S**_0_. A right-handed orthonormal system {**t**_1_, **t**_2_, **t**_3_} can then be defined by 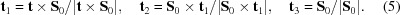
Now, for a spot located at pixel coordinates *x*, *y* in the *COLSPOT* step a scattering vector **S** can be calculated, 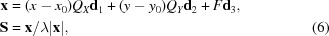
which intersects the powder plane at unit distance at coordinates 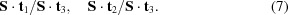
In the ideal case the scattering vectors mark a set of concentric rings: the powder pattern. The centre of the rings should be at the tip of the incident beam, which is the image centre. Often the centre of the powder rings is instead found to be offset from its ideal position, which can be interpreted to result from an incorrect value for the origin of the detector coordinate system **D**_0_.

### 3.5. *IDXREF*   

For each snapshot this step tries to find a crystal lattice that explains the observed diffraction spots by assigning indices to them and refines all parameters. The step returns a list of the successfully processed images and the indexed spots as well as the corresponding diffraction parameters. These parameters are refined for accurate prediction and integration of all reflections that are expected to occur in each snapshot.

Short real-space lattice vectors are determined by the method of Steller *et al.* (1997 ▶). Extraction of a reduced cell, Bravais lattice determination and indexing of the observed spots is performed as described by Kabsch (1993 ▶). The computational methods were taken from *XDS*, with the exception of the refinement procedure, which had to be adapted for handling stills as well, where the concept of a rotation axis has no meaning. This is achieved by using a different refinement target function. Instead of a rotation angle about a fixed camera axis, the smallest angle is used by which a reciprocal-lattice point could reach the Ewald sphere by some unrestricted rotation. A closed analytical expression for this angle is derived below and it is subsequently shown how it is used in the refinement procedure.

#### 3.5.1. Rotating a reciprocal-lattice point to the Ewald sphere   

We would like to find the smallest rotation that moves a reciprocal-lattice point onto the Ewald sphere. Let 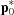 denote any arbitrary reciprocal-lattice vector and **S**_0_ denote the incident-beam wavevector of length 1/λ (λ is the wavelength) pointing from the X-ray source towards the crystal. Diffraction occurs along the wavevector **S** when the crystal is rotated so that 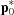 is changed into p* on the Ewald sphere satisfying the Laue equations
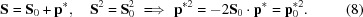
The distance vector 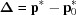 depends on the rotation used and thus is not unique.

Extending earlier work (Kabsch, 1988*b* ▶), a unique rotation can be found for a given **S**_0_, 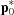 that yields the shortest distance vector 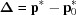, provided 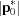 < 2|**S**_0_| and 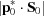 < 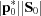.

The unique rotation can be obtained by minimizing the function 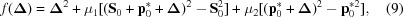
where the two constraints on the solution are enforced by the Lagrange multipliers μ_1_, μ_2_. At the minimum the gradient of *f*(**Δ**) must vanish and the matrix of second derivatives must be positive definite, 
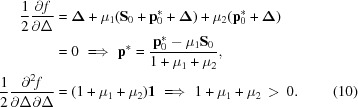
The Lagrange multipliers are adjusted so that the constraints are satisfied. At a vanishing gradient of *f*, the Laue equations are satisfied if 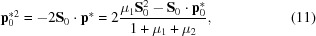
which implies 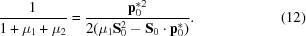
Conservation of the length of the reciprocal-lattice point implies that 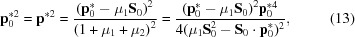
which leads to a quadratic equation for μ_1_, 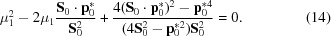
The solution is 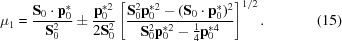
From 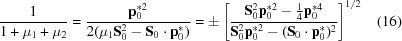
it can be concluded that the positive sign refers to the minimum of **Δ** ^2^, while the negative sign corresponds to the maximum distance from the Ewald sphere. Using the abbreviations 
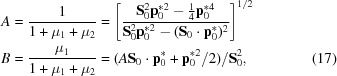
the closest point p* on the Ewald sphere reachable by a rotation of 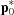 is 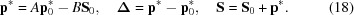
Obviously, the vectors **Δ**, **S**, p* all lie in the plane defined by 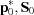. We conclude that 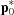 reaches the Ewald sphere at p* by the shortest path if it is rotated about an axis parallel to the plane normal and passing through the origin of reciprocal space. The rotation axis is identical to the ‘β-axis’ (Schutt & Winkler, 1977 ▶) defined for each reflection to be perpendicular to both the incident-beam and diffracted-beam wavevectors.

In the case of the rotation method the crystal is forced to rotate about a fixed axis **m**_2_ which has a component ζ on the ‘β-axis’. ζ is called the reflecting-range expansion factor (Kabsch, 2010*b* ▶) because the angle to rotate a reflection to the Ewald sphere about **m**_2_ increases (by 1/ζ).

Finally, the angular deviation of the reciprocal-lattice point 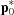 from the Ewald sphere can be defined as the vector 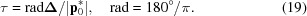



#### 3.5.2. Initial refinement   

For each image, let *j* enumerate the *n* strong spots with observed centroids *X_j_*′, *Y_j_*′, detector segment identifying number *s_j_* and reflection indices *h_j_*, *k_j_*, *l_j_*. To each *j*, we denote the reciprocal-lattice point 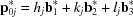, from which a diffracted beam wavevector **S**_*j*_, an angular deviation from the Ewald sphere τ_*j*_ and reflecting-range expansion factors ζ*_j_* can be determined as described in the previous section. With the detector segment recording the strong spot at distance 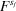, orientation 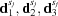 and origin 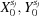, the residuals between the calculated and observed spot centroids are (segment specified in the *laboratory* system)
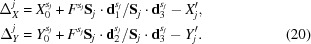
The goal of the refinement procedure is to find the detector parameters, incident-beam direction and unit-cell basis vectors that minimize the target function
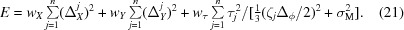
The positional part of the target function pushes the calculated spot positions towards the observed centroids, while the angular part minimizes the angular deviations from the Ewald sphere. The variance for the angular part is expected to increase with the size of the rotation range.

The residuals are expanded to first order in the parameter changes so that *E* becomes a quadratic function of these changes. Minimization then leads to a system of normal equations whose solution is used to update the parameters. During refinement, the cell metric obeys constraints imposed by the lattice symmetry by choosing an appropriate set of free parameters for representing the allowed changes of the basis vectors (details not shown).

The minimization procedure is repeated keeping the same set of weights *w*_*X*_, *w*_*Y*_, w_τ_ until convergence is reached. New weights are then determined so that 
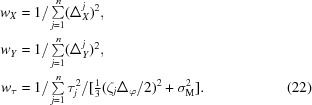
Refinement continues with the new weights until convergence is reached again. The whole refinement procedure is terminated upon convergence of the weights *w*_*X*_, *w*_*Y*_, w_τ_.

### 3.6. *INTEGRATE*   

Starting with the refined diffraction parameters for the successfully indexed data images, this step determines the recorded reflection intensities by two-dimensional profile fitting and saves the results on file for subsequent processing by the *CORRECT* step. No correction factors are applied except for compensating missing parts in the reflection profile owing to overlap with bad pixels or closely neighbouring reflections.

Similar to the integration procedure as implemented in *XDS* (Kabsch, 2010*b* ▶), analysis of each image consists of determination of the reflection spot size and the mosaicity and refinement of the diffraction parameters using the strong spots. This is followed by determination of a two-dimensional reflection reference profile and integration by profile fitting for all expected reflections, including the weak reflections

#### 3.6.1. Mapping image pixels to the Ewald sphere   

As described earlier (Kabsch, 1988*b* ▶, 2010*b* ▶) and implemented in the program *XDS* (Kabsch, 2010*a* ▶), it is useful to represent the intensity distribution in a reflection-specific coordinate system {**e**_1_, **e**_2_, **e**_3_} so that all reflections appear as if they had followed the shortest path through the Ewald sphere and were recorded on the surface of the sphere. This eliminates reflection-specific differences in the intensity profile caused by the oblique incidence of the diffracted beam on a flat detector segment or by crystal rotation around a fixed axis for a rotation data image.

For a reciprocal-lattice point 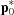 the nearest point **S** on the Ewald sphere is obtained as described above, so that a reflection-specific coordinate system can be defined as 
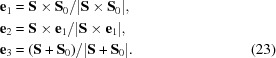
It has its origin at the terminus of the diffracted-beam wavevector **S** and therefore could move depending on the specific path of 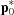 through the Ewald sphere. The unit vectors **e**_1_ and **e**_2_ are tangential to the Ewald sphere, while **e**_3_ is perpendicular to **e**_1_ and p* = **S** − **S**_0_.

Diffraction along wavevector **S**′ in the neighbourhood of **S** is recorded at pixel *X*′, *Y*′ in the detector plane. This pixel is at a distance *D* from the crystal. With the detector at a distance *F*, an orientation **d**_1_, **d**_2_ and an origin *X*_0_, *Y*_0_ of the detector plane, we have 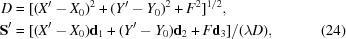
The corresponding coordinates ∊_1_, ∊_2_, ∊_3_ in the reflection-specific system on the Ewald sphere are then 
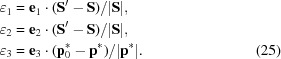
In the case of a rotation image, a reciprocal-lattice point 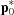 needs a larger angle ∊_3_/|**e**_1_·**m**_2_| to reach the Ewald sphere because the movement is restricted by the fixed rotation axis **m**_2_. The reflecting-range expansion factor ζ = **e**_1_·**m**_2_ corrects for this effect. It is closely related to the reciprocal Lorentz correction factor for rotation images, 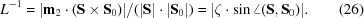
The reflection-specific coordinate system defined above sets up a one-to-one correspondence between the points of a region *R* of the ∊_1_∊_2_ plane tangential to the Ewald sphere and a region *R*′ of the *X*′*Y*′ plane of the detector segment. The Jacobian of the transformation is 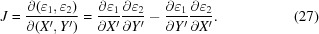
From 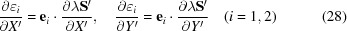
one finds 
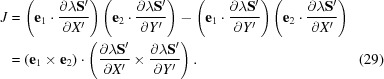
Using 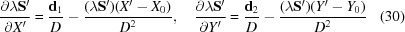
one finds 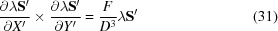
and the Jacobian reduces to the simple expression 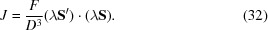
Thus, if the region *R*′ of the *X*′*Y*′ plane of the detector covers a single pixel, the corresponding area *R* in the ∊_1_∊_2_ plane is the mean value of |*J*| times the pixel area, 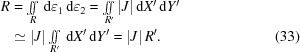



#### 3.6.2. Intensity recorded by a detector pixel   

Because of crystal mosaicity and beam divergence, the intensity of a reflection is smeared around the diffraction maximum. Following earlier work (Kabsch, 1988*b* ▶, 2010*b* ▶), the fraction of the total reflection intensity found in the volume element *d*∊_1_ *d*∊_2_ *d*∊_3_ at ∊_1_, ∊_2_, ∊_3_ is modelled as the product of two functions:
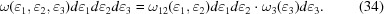
The first function ω_12_(∊_1_, ∊_2_) describes the expected intensity profile of the reflections mapped to their specific ∊_1_, ∊_2_ plane tangential to the Ewald sphere. Initially, Gaussians are assumed with the parameter σ_D_ modelling the combined effects of beam divergence and mosaicity as 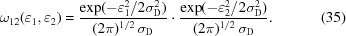
σ_D_ is estimated from the observed variance in intensity of scattered rays for the strong reflections and provides information on the spot width. In a second step the initial Gaussian form for ω_12_ is replaced by the superposition of the profiles of all strong reflections and is used for definition of the final integration region included in profile fitting of all reflections predicted to occur in the diffraction image.

The second function, the rocking curve ω_3_(∊_3_), models the dependency of the reflection intensity on the angular distance from the surface of the Ewald sphere as a Gaussian with the mosaicity σ_M_ of the crystal as the standard deviation. An estimate of σ_M_ is found as described previously (Kabsch, 2010*b* ▶). If τ = rad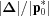 is the angular deviation of the reciprocal-lattice point 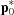 from the Ewald sphere, the fraction of intensity recorded on the image, *i.e.* the partiality of the reflection, is 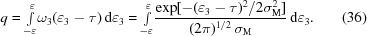
The integration extends over the rotation range Δ_ϕ_ of the spindle during exposure of the image multiplied by the reflecting-range expansion factor ζ, which corrects for the increased path length of the reflection through the Ewald sphere when rotated around a fixed axis, *i.e.* ∊ = ζ·Δ_ϕ_/2. Using the dimensionless variables *t* = τ/2^1/2^σ_M_ and *z* = ∊/2^1/2^σ_M_ the partiality of the reflection can also be given in terms of the error function erf: 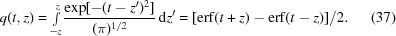
Together with the above expression for the Jacobian *J*, the expected fraction of reflection intensity recorded by pixel *X*′, *Y*′ in the detector plane is, according to our model, 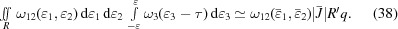
Here, 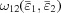 and 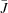 denote mean values in the region *R* of the ∊_1_∊_2_ plane that maps to the region *R*′ of the pixel at *X*′, *Y*′ in the plane of the detector.

### 3.7. *CORRECT*   

After resolving possible indexing ambiguities, the raw intensities of the (reindexed) reflections as obtained from the previous step are scaled and corrected by a modified post-refinement procedure and then saved in the final reflection output file for subsequent structure-solution software packages.

As described below, the new concept of the Ewald offset correction factor as a replacement for partiality links raw intensities to structure-factor amplitudes, rendering classical post-refinement applicable even to still snapshots.

#### 3.7.1. Correction factors   

A reflection intensity 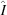, as recorded on a snapshot and returned from the *INTEGRATE* step, is proportional to the ‘true’ intensity *I* (the squared structure-factor amplitude),
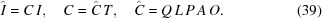
The correction factor *C* consists of a factor 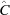 that is not affected by possible indexing ambiguities and a factor *T* that accounts for different scale and resolution fall-off between the snapshots.


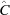
 is a product of factors modelling the Ewald offset correction (*Q*), Lorentz factor (*L*), polarization (*P*), air absorption (*A*) and sensor-thickness correction (*O*). These corrections are functions only of the diffraction parameters of the snapshot that recorded the reflection.

*Ewald offset correction*. The recorded intensities of the reflections of the same image would be directly comparable if they simultaneously obeyed the Laue equations, *i.e.* τ = 0 for all reflections. The idea is to correct the intensity by the estimated decline owing to the angular distance of the reflection from the surface of the Ewald sphere. Using the dimensionless variables *t* = τ/2^1/2^σ_M_ and *z* = ∊/2^1/2^σ_M_ (∊ = ζ·Δ_ϕ_/2) this leads to the definition of the Ewald offset correction 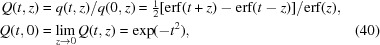
which is well defined even for still images (*z* = 0). *Q*(*t*, *z*) can be calculated for each reflection from the incident-beam wavevector **S**_o_, the reciprocal basis 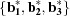, the mosaicity σ_M_ and the oscillation range Δ_ϕ_.

*Lorentz factor*. Assuming an infinitely thin Ewald sphere, the Laue equations can be satisfied for a reciprocal-lattice point by a variation in the wavelength or the direction of the incident beam relative to the crystal. For an ideal crystal the scattered intensity is sharply concentrated around the direction of the diffraction maximum with a solid angle much smaller than the finite aperture of a detector pixel. Consequently, only integrated intensities can be observed: these are related to their squared structure factors by the Lorentz correction. Explicit forms of the corrections are available (see, for example, Zachariasen, 1945 ▶) for all conceivable methods of recording sharp diffraction maxima.

(i) Rotation method. For a fixed wavelength but a variable direction of incidence (accomplished by rotation of the crystal around a fixed axis and a fixed incident beam), the correction factor is
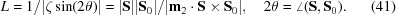
For the special case that the rotation axis is perpendicular to both the incident and diffracted beams, *i.e.* ζ = 1, the Lorentz correction simplifies to 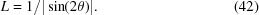
Still snapshots can be considered as a limiting case in which all reflections move infinitesimally through the Ewald sphere along their shortest routes.
(ii) Laue method. For a variable wavelength but a fixed direction of the incident beam, the correction factor is
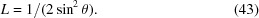

(iii) Powder method. For a fixed wavelength but a variation of the incident beam with two degrees of freedom (accomplished by mosaic crystals), the correction factor is
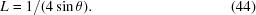

Lorentz correction is always a simple function of resolution and therefore does not affect the agreement among intensities of symmetry-related reflections.

*Polarization*. The intensity of scattering from the crystal is proportional to the polarization factor 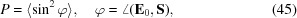
where **E**_0_ denotes the electrical field vector of the incident beam and **S** the diffracted beam wavevector. If **n** denotes the polarization plane normal and *p* the probability of finding the field vector **E**_0_ in this plane, 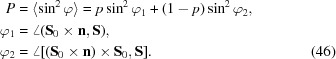
For an unpolarized incident beam, the electrical field vector **E**_0_ is found with equal probability pointing along **S**_0_ × **S** or (**S**_0_ × **S**) × **S**_0_, so that 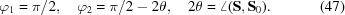
This leads to 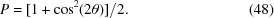
The chosen parametrization allows description of the effect of polarization for most data-collection scenarios (Kahn *et al.*, 1982 ▶). Values for the two parameters **n** and *p* are provided by the user (not refined).

*Air absorption*. The recorded intensity is reduced from its ‘true’ value owing to air absorption of the diffracted beam by the factor 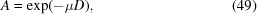
where μ denotes the fraction of intensity loss per millimetre and *D* is the distance (in millimetres) between the crystal and the position of the reflection spot on the detector segment. μ is a wavelength-dependent input constant (not refined).

*Sensor-thickness correction*. The recorded intensity is increased owing to the effect of the oblique incidence of the diffracted beam on a sensor of finite thickness. Let δ denote the thickness of the sensor and κ the fraction of intensity loss per millimetre. If the diffracted beam makes an angle ω with the segment normal, the probability of a photon penetrating the sensor undetected is exp(−κδ/cosω). Thus, the correction 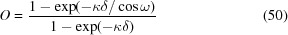
accounts for the oblique incidence of the diffracted beam. Values for δ and κ are provided by the user and are not refined.

*Scaling and temperature factor*. Using the parameters *g* and *B*, the factor 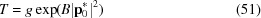
puts the ‘true’ intensity for reciprocal-lattice point 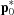 on the same scale as the observed one in the data image. The values of *g* and *B* adjust differences in beam intensity and in crystal disorder and volume in a resolution-dependent way. This correction factor is obtained by comparison of symmetry-equivalent reflections from different images and therefore depends on a consistent indexing choice. For resolving possible indexing ambiguities, correlations between equivalent reflection pairs from different snapshots must be computed (Brehm & Diederichs, 2014 ▶). To manage a potentially large number of snapshots and reflections, an efficient solution was implemented in *nXDS*.

#### 3.7.2. Efficient calculation of correlation factors   

A procedure was developed for calculating correlation factors in which the number of operations is proportional to the total number of recorded reflections of the compared snapshots. The procedure consists of two parts: initialization of the data structures and calculation of the correlation factor between the unique intensity estimates from the compared snapshots.

*Initialization*.

(i) For the given space group and unit-cell parameters all possible unique reflections within a given resolution range are generated. A prime number is determined that is slightly larger than twice the number of possible unique reflections and is used to allocate space for a hash table of this size.
(ii) For each possible unique reflection a positive 64-bit integer is constructed from its unique indices and assigned a definite address in the table using the technique of hash-key transformations with quadratic probing to resolve key collisions (Wirth, 1976 ▶).
(iii) For each reflection from the snapshots the unique indices are determined and coded by their hash-table address, which is saved as an auxiliary reflection attribute. Thus, two reflections are symmetry-related only if they have identical hash-table addresses.
(iv) Four auxiliary arrays of the size of the hash table are allocated: two for each data set to be compared. They are needed for calculating unique intensities and their variances for the reflections of the two data sets.

*Correlation*.

(i) Unique intensities and variances are estimated from symmetry-related reflections of the first snapshot by updating the contents of their associated hash addresses in the first two auxiliary arrays.
(ii) For the intensity data from the second snapshot the procedure is repeated, this time updating the second two auxiliary arrays only if there is a positive entry from the first snapshot at the same hash address in the first two auxiliary arrays.
(iii) Pairs of corresponding unique reflection intensities are obtained easily by scanning the second two auxiliary arrays for a positive contents.

Thus, the total number of operations for calculating the correlation factor between one snapshot and all others is only proportional to the total number of recorded reflections of the snapshots.

#### 3.7.3. Indexing alternatives   

For a given space group and unit-cell parameters, a reciprocal cell basis in some reference orientation (Kabsch, 1988*a* ▶) is first defined and serves as a reference basis. As each image is indexed and integrated independently, it often happens that the reflection indices refer to different (reduced) cells. The problem is to find for each image the set of possible reindexing transformations that allow the reflections to be described in terms of a rotated version of the reference basis.

This set of reindexing transformations is determined for each image using the following procedure. From the reciprocal basis vectors used for indexing the reflections of the image in the *INTEGRATE* step, a reduced cell and its reciprocal cell are determined. The three reciprocal vectors thus obtained are considered as reflections that need to be indexed with respect to a rotated version of the reciprocal reference basis. Possible indices of the reduced-cell reciprocal vectors with respect to the reference basis are found by simply testing all possibilities involving indices absolutely smaller than 4. For each assignment of indices a residual error for the best superposition with the reference basis is determined (Kabsch, 1976 ▶, 1978 ▶) and is used as a measure of the quality of the indexing. Index assignments related by symmetry of the reference cell are omitted, so that a list of symmetry-independent interpretations remains. This list is sorted by increasing r.m.s. of the superposition with the reciprocal reference cell. Entries in the list with an r.m.s. larger than some multiple of that of the first item are omitted.

The list may be empty if no reasonable interpretation is found for the basis vectors of the image. In this case the image is omitted from further calculations.

#### 3.7.4. Resolving the indexing ambiguity   

Ideally, only one symmetry-independent solution remains, identified using the above procedure solely by geometrical considerations. However, for merohedral and pseudo-merohedral crystals, where the lattice symmetry is higher than the symmetry of the point group, more than one choice for the reindexing transformation exists. In *nXDS* the indexing ambiguity can be resolved by using one of two methods. Both methods rely on correlation factors between intensities that have been corrected by 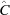 for various effects as described above. Note that the scaling corrections *T* are not needed here.

*Comparison with a reference*. For each snapshot all possible reindexing transformations are tested and the indexing choice yielding the largest correlation factor with the given reference data set is selected. Here, symmetry-equivalent reflection intensities from the snapshot as well as from the reference data are merged separately prior to calculation of the correlation factor. Moreover, an initial scaling factor is determined at little additional computational effort that puts the intensities from each snapshot on the level of the reference.

*Selective breeding*. If no external reference data set is available, a solution is found by a method that is reminiscent of the technique of selective breeding. The method initiates a cyclic procedure with some arbitrary indexing transformation from the list of possibilities assumed by each snapshot. For each cycle the following steps are carried out.

(i) For each snapshot all of its possible indexing choices are tested in succession, with the reindexed reflection intensities treated as a hypothetical reference data set. The mean value of the correlation factors with all other snapshots is calculated, and the running number of the reindexing choice yielding the largest correlation factor is saved. If several choices result in the same value for the correlation maximum, the first one in the list is selected.
(ii) At the end of the cycle the list of optimal indexing choices just determined is used and replaces the previous selection. For some snapshots the new running number of the reindexing choice may differ from that of the previous cycle. If none of the snapshots needs to be assigned to a different indexing choice than before, the cyclic procedure terminates.

This procedure usually terminates within ten cycles. The moderate amount of storage needed is proportional to the number of snapshots and to the size of the hash table. In addition one array is needed that keeps the hash-table address of the unique indices for each reflection of the whole set of images. This speeds up the computation of correlation factors between all pairs of snapshots. Within each cycle these computations can be carried out very efficiently and in parallel by a team of processors.

#### 3.7.5. Scaling   

We assume that all reflections have been consistently indexed. The reflection intensities of each image are still on different scales owing to differences in the intensity of the incident beam or irradiated crystal volume. Determination of the scale factors by the method described here is based on intensity estimates for the unique reflections occurring on each image, 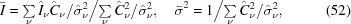
where ν enumerates the symmetry-equivalent reflections and 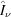 and 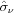 the recorded intensities on the same image and their estimated standard deviations, respectively. 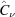 denotes the correction factors as described above. A scaling correction factor for each image is determined by least-squares minimization using common unique reflections with a positive intensity that occur in more than one image.

Let *h* enumerate the *n_h_* different unique indices of the reflections involved in scaling and *l* enumerate the *n_l_* images from which the reflections come. Let *j* enumerate the *n* unique reflections included. To each *j*, we denote intensity 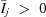, variance 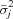, unique reflection index *h_j_* and image *l_j_*. The goal of the scaling procedure is to find factors *g_l_* > 0 and mean intensities *I_h_* > 0 by minimizing the target function (Hamilton *et al.*, 1965 ▶) 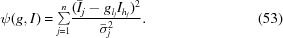
To guarantee success of the solution method for a large set of snapshots and possibly large variations in their scaling factors, the target function is modified by using logarithms, 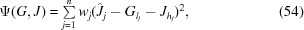
defining 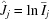, 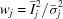, *G_l_* = ln*g_l_* and *J_h_* = ln*I_h_*. This target function is quadratic, with a constant matrix of second derivatives and positive diagonal elements: 
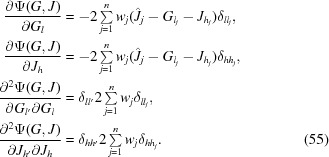
Therefore, unique directional minimizers can be defined by equating the gradients to zero: 
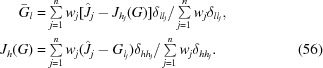
The target function is monotonically reduced by a cyclic procedure of alternating minimizations along the *J* and *G* directions. The procedure is initiated at *G_l_* = 0, *J_h_*(*G* = 0). In each following cycle new scaling factors are found as 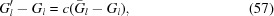
with the step size *c* chosen to maximize the reduction of the target function. Expansion of the quadratic target function at the point 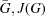 yields 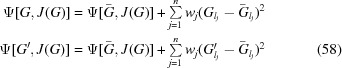
and the resulting reduction in the target function is 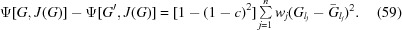
Moving from *G* to *G*′ changes the mean logarithmic intensities by 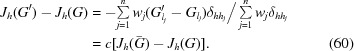
Expansion of Ψ at point *G*′, *J*(*G*′) yields 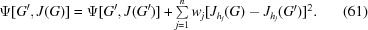
Using the abbreviations 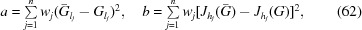
the reduction in the target function at completion of one cycle is 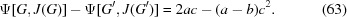
A finite value for the reduction requires *a* > *b*, which leads to an optimal step size *c* = *a*/(*a* − *b*), so that the target function is reduced by Ψ[*G*, *J*(*G*)] − Ψ[*G*′, *J*(*G*′)] = *ac*.

For the gradient, we have 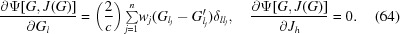
Since the target function is non-negative, the procedure generates a bounded monotonically decreasing sequence converging to the minimum of the target function at vanishing gradient. As shown above, this also implies convergence of the sequence of scaling factors.

Obviously, the solution thus obtained is not unique because the target function does not change its value if one adds an arbitrary constant to the logarithmic scaling factor *G_l_* and subtracts an appropriate constant vector from *J_h_*. This amounts to just changing the common scale of all images in the original problem, which is of no importance as we are only interested in the relative scale.

It may happen that several sets of images exist that are not connected by common measurements. In this case the target function could be thought of consisting of a sum of the same type of target functions, one for each unconnected subset of images. Now there is an arbitrary common factor for each subset. Apparently, the presence of arbitrary common factors for each subset of images does not prevent convergence of the cyclic solution procedure described here.

#### 3.7.6. Post-refinement   

As mentioned above, the diffraction parameters of each image are refined in the *IDXREF* and *INTEGRATE* steps to minimize deviations between the observed and the predicted locations of the strong spots and to minimize their angular distance from the Ewald sphere. The angular part of the target function takes care of the fact that reflections can be visible only if they are close to the Ewald sphere. In addition, the distribution of τ angles thus obtained provides an initial guess for the crystal mosaicity σ_M_. However, the angular part of the initial refinement target cannot account for the fact that very strong reflections can still be observed even if they are farther away from the Ewald sphere than the weaker reflections. This leads to a systematic bias in the initial parameter refinement so that strong reflections will be predicted to be closer to the Ewald sphere than they really are.

These deficiencies can be overcome when all images have been processed and intensity estimates for the recorded reflections are included in the refinement, which explains why this approach has been dubbed ‘post-refinement’. The original idea (Schutt & Winkler, 1977 ▶; Rossmann *et al.*, 1979 ▶; Harrison *et al.*, 1985 ▶; Rossmann, 1985 ▶) is extended here to handle still snapshots as well when fully recorded, measured reflections are not available for comparison and the notion of ‘partiality’ loses its meaning. Its role is assumed by the Ewald offset correction *Q* defined above that is applicable for rotation images as well as stills. The ‘post-refinement’ variant implemented here considers the possible unique reflection intensities as free parameters that are to be refined along with the diffraction parameters of each snapshot (Bolotovsky *et al.*, 1998 ▶).

The goal of the refinement procedure is the minimization of the target function 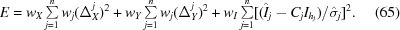
Here again, *j* enumerates the *n* recorded reflections from all snapshots, Δ_*X*_^*j*^, Δ_*X*_^*j*^ the residuals between the calculated and observed spot centroids (see §[Sec sec3.5.2]3.5.2) and 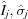 the recorded raw intensity and its standard deviation. If a spot *j* is strong enough so that a centroid could be determined then *w_j_* = 1, otherwise *w_j_* = 0.

Each spot *j* is associated with a reciprocal-lattice point 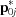 close to the Ewald sphere. For each observation a correction factor *C_j_* can be computed from the diffraction parameters that relates the recorded intensity 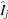 to a unique reference intensity 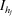, where *h_j_* denote the unique indices of the reciprocal-lattice points 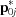.

The target function *E* depends on private parameters for each snapshot and global parameters and constants.

(i) *Private parameters*.**S**_0_, the incident-beam wavevector.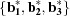, the reciprocal cell basis vectors.*g*, the scaling factor for intensities.*B*, the isotropic temperature factor.σ_M_, the mosaicity.
detector parameters.
(ii) *Global parameters*.*I_h_*, the squared structure-factor amplitudes for the possible unique reflections *h* (up to some global constant irrelevant in this context).
(iii) *Global constants*.**n** and *p*, the polarization plane normal and the degree of polarization.μ, the fraction of intensity loss per millimetre in air.δ, the thickness of the detector sensor.κ, the fraction of intensity loss per millimetre in the sensor.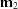 and Δ_ϕ_, the rotation axis and oscillation range (Δ_ϕ_ = 0 for ‘stills’).

Each refinement round starts with the determination of the unique reflection intensities *I_h_*, keeping the current parameter values constant. Minimization of the target function yields 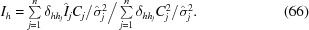
The weights are then calculated as 
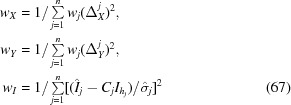
and are kept throughout the refinement round. The whole procedure is terminated upon convergence of the weights *w_X_*, *w_Y_*, *w_I_*.

During a refinement round the diffraction parameters for each snapshot are corrected iteratively to minimize the target function until convergence is reached. The residuals are expanded to first order in the parameter changes so that *E* becomes a quadratic function of these changes. Minimization then leads to a system of normal equations whose solution is used for updating the parameters. The gradients of Δ_*X*_^*j*^, Δ_*Y*_^*j*^ and *C_j_* are computed from analytic expressions (not shown).

Fortunately, these calculations are independent for each snapshot and can be performed in parallel by a team of processors. Moreover, the memory requirements are almost negligible even when the refinement of detector parameters is included.

## 4. Example of data processing with *nXDS*   

As an example to demonstrate the quality of data processed by *nXDS* in comparison to conventional data reduction by *XDS*, 20 000 consecutive rotation images were collected at 100 K from a crystal of a selenomethionine-labelled double mutant of the RNA-processing factor SCAF8 (Becker *et al.*, 2008 ▶). Each image covers a rotation range Δ_ϕ_ of 0.02° and is treated by *nXDS* as a snapshot taken from a randomly oriented crystal. The images were collected on beamline X10SA at the Swiss Light Source, Villigen, Switzerland at a wavelength of 0.9779 Å, slightly above the Se *K* edge. The images were recorded by a PILATUS 6M pixel detector (Dectris AG, Baden, Switzerland) located at 300 mm distance. The crystal has *P*4_3_ space-group symmetry, which is lower than the 422 lattice symmetry, implying a twofold indexing ambiguity.

The processing results are summarized in Table 1 ▶. The upper part refers to the evaluation of the images by *XDS* as conventional rotation data. The lower part shows the corresponding quantities as obtained from *nXDS*. Here, reflections were only included if their Ewald offset correction was larger than 0.7. A total of 356 854 reflections in the resolution range 15–2 Å were integrated by *XDS*, so that for each unique reflection almost eight symmetry-related reflections are available. Because contributions to each reflection are also recorded by adjacent images, it is not surprising that *nXDS* found almost ten times more reflections, which is consistent with the correspondingly higher multiplicity of observations.

After merging symmetry-related reflection intensities one might expect that the mean signal-to-noise ratio 〈*I*/σ(*I*)〉 would come out about the same regardless of whether the images were processed by *XDS* or *nXDS*. This is not the case: the mean signal-to-noise ratio is higher by a factor of almost three for the results from *XDS*. The lower accuracy of *nXDS* presumably results from two-dimensional instead of three-dimensional profile fitting and the lack of other corrections not carried out yet by this version of *nXDS*. The nearly perfect correlation factors CC_1/2_ (Karplus & Diederichs, 2012 ▶) between intensities of symmetry-related reflections obtained from processing by both programs reflect the excellent quality of the data images. The presence of anomalous scatterers is clearly indicated in both processing results by the highly significant value for the anomalous correlation CC_ano_. Finally, the reflection intensities obtained from both programs are in excellent agreement, showing a correlation coefficient of 98%. 

Data processing was carried out by a 12-core machine with 16 GB memory running under Linux. Making use of the hyperthreading capability, up to 24 threads were employed for processing the images stored on a local disk. Elapsed wall-clock times for each step are listed in Table 2 ▶. *COLSPOT* uses a very fast spot-finding procedure but spends most of its time waiting for the next image to arrive. On average only three out of 24 threads were active. *COLSPOT* is a time-consuming step in *nXDS* because each image had to be analyzed for diffraction spots since the knowledge that the images comprise a rotation data set was not used. In contrast, *XDS* only requires spots from a small fraction of the images for recognizing the crystal lattice and for accurate refinement of the diffraction parameters. For the same reason, the *IDXREF* step in *nXDS* takes much longer than in *XDS*. In the *INTEGRATE* step *nXDS* is somewhat faster than *XDS* because of the reduced overhead in control when only single images are involved. Compared with *XDS*, the *CORRECT* step of *nXDS* takes longer because of the much larger number of reflections. Furthermore, additional computations are required by the selective breeding procedure when no reference data set is available. According to Table 2 ▶, the breeding procedure required about 14 min to resolve the twofold indexing ambiguities for 20 000 snapshots and a total of 3.4 million reflections.

Details of the procedure are shown in Table 3 ▶ as the number of misfitting snapshots. In the first two generations both indexing alternatives are nearly randomly distributed. A small fluctuation towards one choice builds up in the third generation and quickly dominates the population. After five generations a homogeneous population is obtained.

Phasing of single anomalous diffraction data was performed for the *XDS* and *nXDS* processed data sets using the *SHELXC*/*D*/*E* program suite (Sheldrick, 2010 ▶). The results are summarized in Table 4 ▶. In brief, the marker-atom structure factors were estimated from pre-merged data using *SHELXC*. Subsequently, the selenium substructure was determined in a search of 100 trials for ten putative sites while applying a high-resolution cutoff at 2.5 Å. For both data sets, *SHELXD* identified 14 positions, of which seven showed significantly higher occupancy when compared with the less significant positions. This is in good agreement with the expected eight selenium positions (Becker *et al.*, 2008 ▶). Phases were further improved by density modification using *SHELXE*; eight sites were refined to significant occupancy and the phases obtained resulted in excellent electron-density maps with high pseudo-free correlation coefficients CC_free_ (Table 4 ▶) and the correct enantiomorphic setting. Peak heights at the eight heavy-atom sites are highly significant and are well above the largest noise peak. Although phasing for both data sets was unambiguous, the data set processed with *nXDS* showed slightly lower correlation coefficients, Patterson figures of merit (PATFOM) and heavy-atom peak heights.

## 5. Conclusion   

This study describes a new approach for processing a large set of snapshots from randomly oriented crystals that does not rely on the Monte Carlo method of integration. Instead, the concept of the Ewald offset correction factor was devised to overcome difficulties arising from the use of partiality for modelling reflection intensities recorded by snapshots. The new approach has been implemented in the program *nXDS* that has borrowed many ideas and routines from the rotation data-processing package *XDS* as well as from the powerful post-refinement technique that has been in widespread use for several decades.

The implemented Ewald offset correction relies on a Gaussian model for the rocking curve, assuming a sufficiently large crystal whose shape transform can be ignored. In this case the exact functional form of the curve is not critical for reflections sufficiently close to the Ewald sphere. In the test data set, a reflection was only included if its Ewald correction factor was larger than 0.7.

As shown for the test case with fine-sliced rotation images of excellent quality, *nXDS* delivers results almost approaching those obtained by *XDS* and is able to retrieve the anomalous signal from a selenomethionine-labelled protein crystal. The source of the lower accuracy of *nXDS* is not yet clear. It could result from two-dimensional instead of three-dimensional profile fitting and the omission of information from weak contributions to reflections further away from the Ewald sphere that are used only by *XDS*. In fact, a small improvement in overall data quality by 0.9% in 〈*I*/σ(*I*)〉 and 2.9% in CC_ano_ was observed upon the inclusion of weaker contributions when the minimum required Ewald offset correction was lowered from 0.8 to 0.7.

So far, no ‘real’ FEL data have been processed by *nXDS*. These data typically vary for each snapshot in wavelength, bandwidth and crystal parameters. Although *nXDS* allows some of these to change for each snapshot, program modifications are likely to become necessary when the incident-beam bandwidth can no longer be substituted by a mean wavelength or if the shape transform of the crystals cannot be ignored. Presently, *nXDS* can only accept images that *XDS* can read. Work is in progress to adapt the package to also handle the detectors used at FEL beamlines and to make *nXDS* and its documentation available from the internet.

## Figures and Tables

**Table 1:** Comparison of data processing with *XDS* and *nXDS*

Resolution (Å)	15–2	15–6	6–4	4–2.1	2.1–2
*XDS*					
Reflections	356854	12414	31981	264002	48457
Multiplicity	7.8	7.7	7.8	7.8	7.7
〈*I*/σ(*I*)〉	38.6	90.9	83.3	35.6	12.2
CC_1/2_ (%)	100.0	100.0	100.0	100.0	99.2
CC_ano_ (%)	76	98	96	71	34
*nXDS*					
Reflections	3409453	56009	231474	2610196	511774
Multiplicity	74.6	37.9	57.4	76.8	82.7
〈*I*/σ(*I*)〉	16.9	24.9	30.2	16.8	6.7
CC_1/2_ (%)	99.9	99.8	99.9	99.8	97.2
CC_ano_ (%)	41	84	72	38	18

**Table 2:** Wall-clock times (s) for processing with *XDS* and *nXDS*

Step	*XDS*	*nXDS*	Comments
*XYCORR*	1	1	
*INIT*	172	124	
*COLSPOT*	82	1864	I/O limited
*POWDER*	—	7	
*IDXREF*	3	1206	
*INTEGRATE*	2022	1704	
*CORRECT*	24	43	Using a reference
		808	Using selective breeding

**Table 3:** Consistent indexing by selective breeding

Generation	Replaced misfits
1	10045
2	9740
3	6380
4	114
5	0

**Table 4:** Phasing statistics

	*XDS*	*nXDS*
*SHELXD*		
Correct solutions for 100 trials	100	96
PATFOM	21.41	14.38
CC_all_ (%)	56.07	38.31
CC_weak_ (%)	34.51	21.99
*SHELXE*		
CC_free_ (%)	71.53	62.25
Heavy-atom peaks (map units σ)		
Strongest (σ)	44	34
Weakest (σ)	19	13
Average (σ)	36	27
Highest noise (σ)	10	8
